# Cigarette Smoke Modulates Repair and Innate Immunity following Injury to Airway Epithelial Cells

**DOI:** 10.1371/journal.pone.0166255

**Published:** 2016-11-09

**Authors:** Gimano D. Amatngalim, Winifred Broekman, Nadia M. Daniel, Luciën E. P. M. van der Vlugt, Annemarie van Schadewijk, Christian Taube, Pieter S. Hiemstra

**Affiliations:** Department of Pulmonology, Leiden University Medical Center, Leiden, The Netherlands; Cedars-Sinai Medical Center, UNITED STATES

## Abstract

Cigarette smoking is the main risk factor associated with chronic obstructive pulmonary disease (COPD), and contributes to COPD development and progression by causing epithelial injury and inflammation. Whereas it is known that cigarette smoke (CS) may affect the innate immune function of airway epithelial cells and epithelial repair, this has so far not been explored in an integrated design using mucociliary differentiated airway epithelial cells. In this study, we examined the effect of whole CS exposure on wound repair and the innate immune activity of mucociliary differentiated primary bronchial epithelial cells, upon injury induced by disruption of epithelial barrier integrity or by mechanical wounding. Upon mechanical injury CS caused a delayed recovery in the epithelial barrier integrity and wound closure. Furthermore CS enhanced innate immune responses, as demonstrated by increased expression of the antimicrobial protein RNase 7. These differential effects on epithelial repair and innate immunity were both mediated by CS-induced oxidative stress. Overall, our findings demonstrate modulation of wound repair and innate immune responses of injured airway epithelial cells that may contribute to COPD development and progression.

## Introduction

Smoking has been shown to increase epithelial inflammation and injury, and has been suggested to disrupt the host defense function of the airway epithelium [1, 2]. These effects may be highly relevant for our understanding of the development of smoking-induced lung diseases [3], including chronic obstructive pulmonary disease (COPD), an inflammatory lung disorder that is characterized by a progressive and irreversible obstruction of airflow [4]. Changes in the airway epithelium resulting from exposure to smoke are early and key events in the development and progression of COPD [5, 6]. Airway epithelial cells, which line the surface of the respiratory tract, normally function as the first host defense barrier against respiratory pathogens [2]. However, extensive epithelial injury, for instance caused by cigarette smoking, respiratory pathogens and inflammation, may lead to disruption of the epithelial barrier integrity and cell death [7–9]. Upon injury, a rapid wound repair process is initiated during which airway epithelial cells produce innate immune mediators to enhance host defenses at the wounded area [10]. These repair responses are essential for restoration of the barrier function of the epithelium, and subsequent regeneration of a pseudostratified layer of epithelial cells. However, the repair process might be altered directly by CS exposure or indirectly by CS-induced inflammation, and this modulation of repair might contribute to COPD development and progression by promoting epithelial remodeling and persistent airway inflammation.

The direct effects of CS on wound repair of airway epithelial cells have been primarily studied by applying an aqueous extract of CS on undifferentiated submerged cultures of airway or alveolar epithelial cell lines or primary airway epithelial cells [7, 11, 12]. However, to gain more insight in the effect of smoking on airway epithelial repair, further research is required using conditions that better reflect the local conditions in lungs of smokers. Air-liquid interface cultures of mucociliary differentiated primary bronchial epithelial (ALI-PBEC) represent a widely accepted model to investigate airway epithelial cell functioning in lung diseases [2, 5]. These cultures are highly similar to the airway epithelium of the small and large conducting airways, and display a pseudostratified morphology including ciliated, secretory, intermediate columnar and basal cells (BCs) [13, 14]. We have used exposure of ALI-PBEC cultures to whole CS to better mimic smoke exposure *in vivo* [9, 15]. Using this model, we have previously shown epithelial injury and transient disruption of the epithelial barrier integrity upon acute exposure to whole CS [9]. This response was accompanied by epidermal growth factor receptor (EGFR)-mediated expression of innate immune mediators, including expression of the neutrophil chemoattractant C-X-C Ligand 8 (CXCL8, or IL-8) and selective expression of the antimicrobial protein Ribonuclease 7 (RNase 7) by BCs present in ALI-PBEC cultures. These findings provided important evidence for a dual function of airway BCs in epithelial repair and innate immunity that requires further investigation [16]. Especially, the influence of CS on the dual function of airway epithelial BCs in mediating wound repair and innate immunity is of interest in view of the development of smoking related diseases such as COPD.

In the present study we examined the effect of epithelial exposure to whole CS on repair and induction of innate immune responses by wounded ALI-PBEC cultures. Epithelial injury was induced by disrupting the epithelial barrier integrity, via disruption of cell junctions or via mechanical wounding of epithelial layers. EGFR-induced innate immune responses were examined by determining the expression of the BC-specific mediator RNase 7 and the luminal airway epithelial cell- and BC-expressed chemokine IL-8. In addition, the role of BCs in wound repair after mechanical injury was determined by assessment of the number of BCs at the wound edge. Moreover, we studied the contribution of oxidative stress and EGFR signal transduction to wound repair and innate immune responses.

## Materials and Methods

### ALI-PBEC and whole CS exposure model

Primary bronchial epithelial cells (PBEC) were isolated from macroscopically normal lung tissue obtained from patients undergoing resection surgery for lung cancer at the Leiden University Medical Center. Use of such lung tissue that became available for research within the framework of patient care was in line with the “Human Tissue and Medical Research: Code of conduct for responsible use” (2011) (www.federa.org), that describes the no-objection system for coded anonymous further use of such tissue. PBEC were cultured and differentiated at the air-liquid interface as previously described [9, 17]. In short, cells at passage 2 were seeded at a density of 40.000 cells/0.9 cm^2^ on 0.4 μm pore sized semi-permeable transwell membranes (Corning Costar, Cambridge, MA, USA) that were coated with a mixture of 30 μg/ml PureCol (Advanced BioMatrix, San Diego, CA, USA), 10 μg/ml bovine serum albumin (BSA) (Sigma-Aldrich, St. Louis, MO, USA) and 10 μg/ml fibronectin (isolated from human plasma) in PBS. Cells were cultured in Bronchial epithelial growth medium (BEGM) (Lonza, Verviers, Belgium) and Dulbecco's modified Eagle's medium (DMEM) (Gibco, Bleiswijk, The Netherlands) (1:1 mixture) containing 1 mM Hepes (Lonza) and supplemented with SingleQuot supplements and growth factors according to the manufacturer’s instructions (bovine pituitary extract [BPE], hydrocortisone, human epidermal growth factor [hEGF], epinephrine, transferrin, insulin, T3 and retinoic acid; all from Lonza), and additional 15 ng/ml retinoic acid (Sigma-Aldrich), 1 mg/ml BSA (Sigma-Aldrich), 100 U/mL penicillin and 100 μg/ml streptomycin (Lonza). Cells were first cultured in submerged conditions until confluence, followed by air-exposed culturing during 2–3 weeks to allow mucociliary differentiation. Exposure of ALI-PBEC to whole cigarette smoke (CS) was done according to a previously described model [9]. In this model, ALI-PBEC were placed in exposure chambers and exposed to air (control) or whole cigarette smoke (CS) derived from one cigarette (3R4F reference cigarettes, University of Kentucky, Lexington, KY, USA) during a period of 15 minutes. Following exposure, the culture medium was refreshed.

### Calcium switch assay

The effect of disruption of the epithelial barrier integrity was examined using the calcium switch assay [18]. In brief, ALI-PBEC were incubated for 15–30 minutes with 700 μL of calcium-free minimum essential medium (Gibco) that was added at the apical side and 1000 μL in the basolateral compartment to deprive cells of calcium to disrupt cell-cell contacts. Epithelial barrier disruption was determined by measuring the trans-epithelial electrical resistance (TEER) using MilliCell-ERS (Millipore, Bedford, MA, USA). After a complete loss of the barrier integrity, the apical medium was removed and cells were exposed to air or CS. Following exposure, the basal medium was replaced with calcium-containing culture medium with growth factors to allow reformation of junctions.

### Wound healing assay

Wound healing assays were performed according to a previously described protocol [7], adapted for use in ALI-PBEC. In brief, the apical side of ALI-PBEC cultures was washed with PBS, and cells were starved for growth factors overnight in starvation medium (supplemented BEGM:DMEM without BPE and hEGF). 500 μL PBS was added to the apical surface of ALI-PBEC to facilitate mechanical injury, which was induced by scraping the cell layer with a sterile Pasteur pipette with a soft tip, creating a wound with a diameter of 3 mm. After wounding, the apical surface of the cultures was washed with 200 μL PBS to remove cellular debris.

In designated experiments, 10 mM of N-acetylcysteine (NAC) (Sigma Aldrich) was used to determine the role of oxidative stress. To investigate EGFR and ERK signaling, cells were incubated with AG1478 (EGFR tyrosine kinase inhibitor) or U0126 (MEK1/2 inhibitor) (both Calbiochem, Darmstadt, Germany). After wounding, ALI-PBEC cultures were exposed to whole CS, and culture medium was replaced by fresh starvation medium, including additional inhibitors as indicated. For live imaging experiments, images of wounded ALI-PBEC were acquired using a Leica DM16000 phase-contrast light microscope (Leica Microsystems, Wetzlar, Germany), collecting digital images of the wound every 15 minutes up to 48 hours. During this period, cells were placed inside a micro cell incubator at 37°C in a 7.5% CO_2_ humidified atmosphere. The acquired images were used to create a time-lapse movie. For other wound healing experiments, digital images were collected on a digital camera connected to an inverted phase-contrast light microscope using Cell Sense Entry imaging software (both Olympus, Tokyo, Japan), at time 0, 6, 24 and 48 h after wounding. The surface of the wound area was measured using Photoshop CS6 (Adobe, San Jose, California, USA) in order to assess remaining wound size and wound closure rates.

### Immunofluorescence confocal imaging

Immunofluorescence staining of wounded ALI-PBEC was conducted as previously described [9]. Cells were stained with a monoclonal anti-rabbit p63 antibody (ab124762, Abcam, Cambridge, UK) (1:100) to detect BCs, and DAPI to stain all nuclei. Z-stack images of the wound edge were made using a Leica TCS SP5 confocal inverted microscope (Leica Microsystems, Wetzlar, Germany) and processed using the Leica Application Suite Advanced Fluorescence software (LAS AF; Leica Microsystems). Five random images of air and CS-exposed wounded ALI-PBEC were used from independent donors to determine the number of p63^+^ cells. The percentage of p63^+^ nuclei was determined at the leading wound edge and at a randomly selected unwounded area. Moreover, the average number of p63^+^ cells at the wound edge was calculated per 400 μm, and the internuclear distance between a p63^+^ cell at the leading wound edge and its first adjacent p63^+^ cell was quantified in approximately 20–30 nuclei per image. Further explanation of this method is provided in S2 Fig.

### Quantitative real-time PCR

RNA extraction, cDNA synthesis and quantitative real-time PCR (qPCR) was conducted as described previously [9]. mRNA expression was examined for the genes described in S1 Table. Relative gene expression compared to reference genes *ATP5B* and *RPL13A* was calculated according to the standard curve method. Reference genes were selected using the “Genorm” software [19].

### Western blot

Western blot analysis of EGFR and ERK1/2 phosphorylation was done as previously described [9]. The following primary antibodies were used: rabbit monoclonal Ab EGFR #D38B1 (1:1000), rabbit polyclonal phospho-EGFR #2234, rabbit polyclonal ERK1/2 #9102 (1:1000), and rabbit polyclonal phospho-ERK1/2 #9101 (all Cell Signalling, Leiden, The Netherlands). Protein bands were quantified by densitometry using ImageJ software (National Institutes of Health, Bethesda, MD, USA).

### ELISA

Secretion of IL-8 (R&D, Minneapolis, MN, USA) was determined according to the manufacturer’s protocol.

### Statistics

Data were analyzed using GraphPad Prism 6.0 (GraphPad Inc., La Jolla, CA, USA). Statistical tests used for data analysis were 2-way ANOVA, with post-hoc Bonferroni correction for multiple analyses. Differences with a p-value < 0.05 were considered as statistically significant.

## Results

### Cigarette smoke delays barrier recovery and enhances innate immune responses by ALI-PBEC

**Fig 1:**
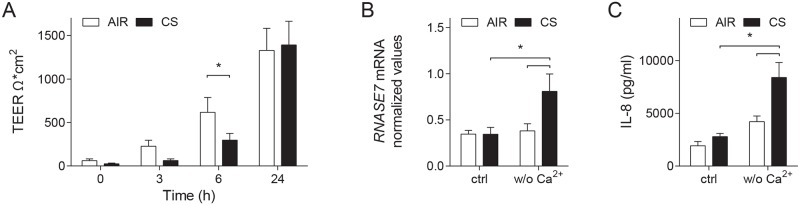
Effects of CS on airway epithelial barrier recovery and innate immunity. Barrier integrity in ALI-PBEC was disrupted using calcium depletion, and cells were subsequently exposed to air or CS. (A) The trans-epithelial electrical resistance (TEER) was subsequently measured at 0, 3, 6, and 24 h after exposure to assess loss and recovery of barrier integrity in air- and CS-exposed cultures. TEER values in ohm (Ω). n = 7 independent donors. (B) At 24 h, mRNA expression of *RNASE7* was assessed in ALI-PBEC that were incubated with calcium-depleted medium (w/o Ca^2+^) versus control medium (ctrl), and subsequently exposed to either air or CS and further incubated in calcium containing medium. Normalized mRNA expression compared to *RPL13A* and *ATP5B* is depicted in the graph. n = 4 independent donors. (C) Secretion of IL-8 in the basal culture medium was assessed by ELISA. n = 5 independent donors. Data are shown as mean; error bars represent SEM; experiments were conducted using duplicate exposures in all donors, * p < 0.05.

We first used a calcium switch assay to determine the effect of CS on recovery of the airway epithelial barrier, and to explore the importance of the loss of barrier integrity for the induction of RNase 7 and IL-8. In this assay, calcium-depleted culture medium was applied at the apical surface and basal compartment of ALI-PBEC, resulting in a complete impairment of the airway epithelial barrier integrity as determined by measuring the trans- epithelial electrical resistance (TEER). Subsequently, cells were exposed to CS or air as negative control. The effect of CS on barrier recovery was determined at different time points, and induction of innate immune responses was assessed at 24 h. Both air- and CS-exposed ALI-PBEC displayed complete recovery of the airway epithelial barrier integrity 24 h after barrier disruption (Fig 1A). However, CS exposure caused a delay in this recovery at 6 h after exposure, which was significantly different compared to air-exposed cells. Assessment of RNase 7 mRNA expression demonstrated a significantly higher expression in CS-exposed ALI-PBEC incubated with calcium-depleted medium (Fig 1B). In control cultures, we did not detect CS-induced expression of RNase 7 at 24 h; in a previous study we also no longer detected CS-induced expression of RNase 7 at this time point [9]. Similar to RNase 7, we observed enhanced secretion of IL-8 in the basal medium upon barrier disruption and CS exposure (Fig 1C). Overall, these findings suggest that CS delays restoration of epithelial barrier function following calcium deprivation, while further increasing innate immune responses.

### Cigarette smoke delays repair and further increases RNase 7 expression in ALI-PBEC

**Fig 2:**
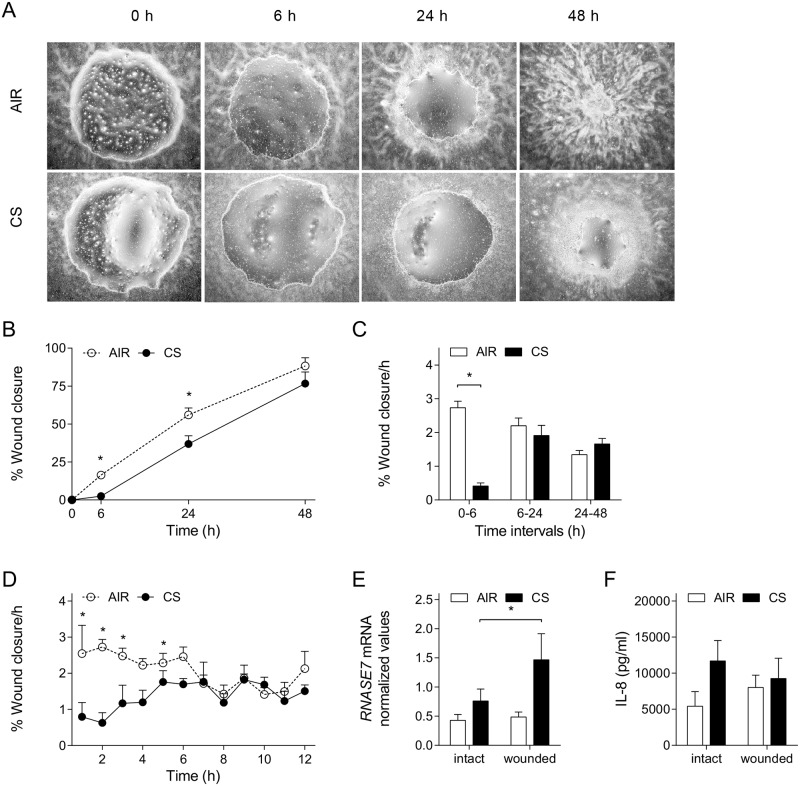
Effect of CS on airway epithelial wound healing and innate immunity. ALI-PBEC were mechanically injured and subsequently exposed to air (control) or whole cigarette smoke (CS). (A) Phase-contrast light microscopy images were made of air- and CS-exposed ALI-PBEC at 0, 6, 24 and 48 h after exposure. (B) Wound closure is shown in percentage in air- versus CS-exposed cells and (C) wound closure rate in percentage per hour at different time intervals was calculated. n = 8 independent donors. (D) Wound closure rates per hour in air- and CS-exposed ALI-PBEC up to 12 h after exposure were determined using live imaging. n = 7 independent donors. (E) *RNASE7* mRNA expression was determined in intact or wounded ALI-PBEC exposed to air or CS, at 6 h after exposure. Values shown represent normalized mRNA expression compared to *RPL13A* and *ATP5B*. n = 7 independent donors. (F) IL-8 secretion was determined in the basal culture medium. n = 9 independent donors. Data are shown as mean; error bars represent SEM; experiments were conducted in duplicate i, * p < 0.05.

To further examine epithelial repair and induction of innate immune responses in wounded airway epithelial cells, we used a wound healing model in which ALI-PBEC cultures were mechanically injured by applying circular wounds. In this model, ALI-PBEC displayed intrinsic wound healing, and full wound closure was observed within approximately 48 h, as measured by live imaging (S1 Fig). Whole CS exposure directly following epithelial injury impaired wound healing of ALI-PBEC during the first 24 h after exposure (Fig 2A and 2B), with significantly decreased wound closure rates during the first 6 h after CS exposure, but not at later time intervals (Fig 2C). Live imaging experiments further demonstrated impaired wound repair at early time points, with recovery of wound closure rates approximately 6 h after CS exposure (Fig 2D).

Next, we determined the effect of CS exposure following mechanical injury on mRNA expression of RNase 7 and protein secretion of IL-8. Comparison between intact and wounded cultures demonstrated significant higher CS-induced mRNA expression of RNase 7 in wounded ALI-PBEC at 6 h after exposure (Fig 2E). In contrast, we did not observe such an effect on IL-8 protein secretion (Fig 2F). Taken together, these findings further demonstrate impairment of epithelial repair upon CS exposure, which was accompanied by induction of RNase 7, but not of IL-8.

### p63^+^ cells at the wound edge are increased in CS exposed ALI-PBEC

**Fig 3:**
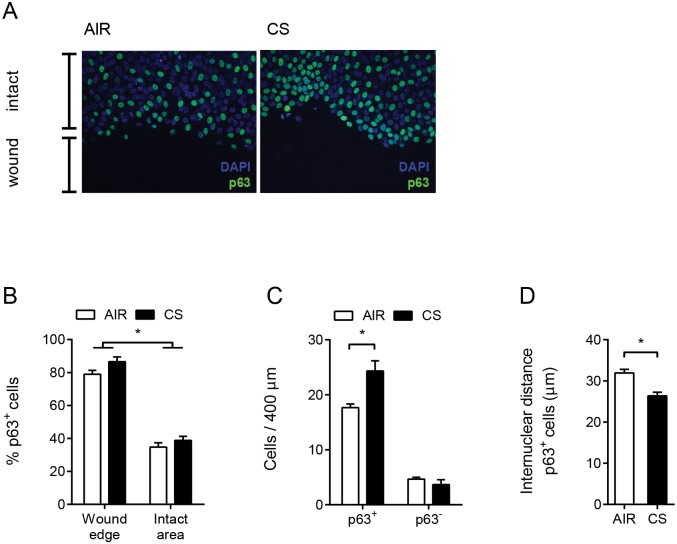
p63^+^ cells at the wound edge of ALI-PBEC. (A) Immunofluorescence staining for p63 (green) and nuclei (blue (DAPI)) of mechanically injured ALI-PBEC. (B) Percentage of p63^+^ cells at the first line of cells directly at the wound edge or in intact areas, in air- versus CS-exposed cells. (C) Number of p63^+^ cells and p63^-^ cells at the wound edge per 400 μm length of wound edge, in air- versus CS-exposed cells. (D) Internuclear distance in μm between p63^+^ cells located directly at the leading wound edge and the first adjacent p63^+^ cell. All graphs: data are shown as mean; error bars represent SEM, experiments were conducted in duplicate, n = 3 independent donors, * p < 0.05.

Previously, we reported cell-type specific expression of RNase 7 by BCs in response to CS, whereas expression of IL-8 was observed in both luminal cells and BCs [9]. The selective increase in RNase 7 expression in CS-exposed wounded cells suggests that CS in particular affects the activity of BCs in wounded ALI-PBEC. Therefore, we next examined the contribution of BCs to wound repair of ALI-PBEC. This was determined by assessing the number of cells at the wound edge that stained positive for the nuclear BC-marker p63 (Fig 3A and S2 Fig). The majority of cells (approximately 80%) directly located at the wound edge stained p63^+^, which was a significantly higher proportion compared to intact areas of the same culture (appr. 35%) (Fig 3B). In CS-exposed cultures, p63^+^ cells appeared to accumulate in higher numbers at the wound edge (Fig 3C). Moreover, we observed in CS-exposed cells significant smaller internuclear distances between p63^+^ cells located at the leading wound edge and located directly adjacent to the wound edge (Fig 3D and S2 Fig for explanation of the method). These observations suggest that CS impairs spreading and migration of BCs, a process that is important especially in the initial phase of wound repair. Collectively, these findings suggest that CS not only affects the innate immune function of BCs during repair, as shown by increased expression of RNase 7, but also affects the wound repair activity.

### Cigarette smoke differentially affects wound repair and innate immune responses through oxidative stress

**Fig 4:**
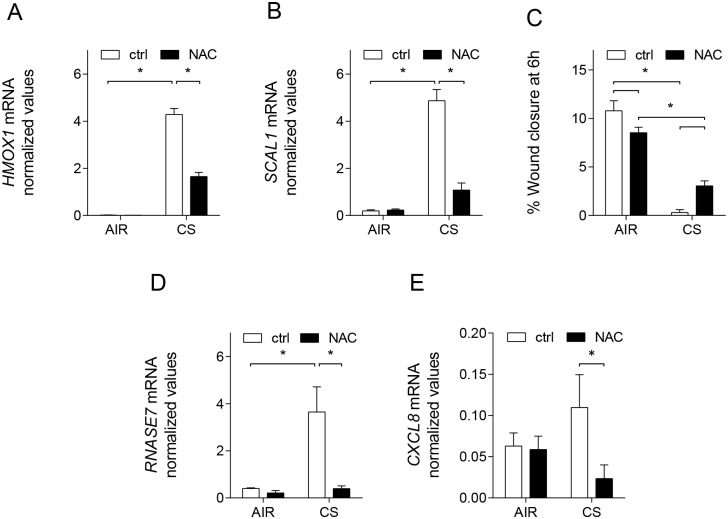
Role of CS-induced oxidative stress in modulating airway epithelial repair and innate immunity. Wounded ALI-PBEC were pre-incubated with NAC (10 mM) and subsequently exposed to air or CS. mRNA expression of the oxidative stress-induced genes (A) *HMOX1* and (B) *SCAL1* was determined 6 h after exposure. Values shown represent normalized mRNA expression compared to *RPL13A* and *ATP5B*. n = 3 independent donors, * p < 0.05. (C) Wound closure in presence or absence of NAC (10 mM) 6 h after wounding, in air- versus CS-exposed cells. Data are shown as percentage wound closure compared to t = 0 h. n = 7 independent donors. (D) mRNA expression of *RNASE7* and (E) *CXCL8* was assessed in wounded ALI-PBEC incubated with NAC (10 mM), at 6 h after exposure to air or CS. Data are shown as normalized mRNA expression compared to *RPL13A* and *ATP5B*. n = 3 independent donors. In all graphs data are shown as mean; error bars represent SEM; experiments were conducted in duplicate; * p < 0.05.

To understand the mechanism of CS-mediated modulation of wound repair and innate immune responses we next examined the role of oxidative stress. The presence of oxidative stress upon CS exposure was demonstrated indirectly by showing CS-induced mRNA expression in wounded ALI-PBEC of heme oxygenase (decycling) 1 (*HMOX1*) and smoke and cancer-associated IncRNA-1 (*SCAL1*), both target genes of the oxidative stress-dependent Nrf2 pathway [20, 21]. This induction by CS was blunted by treatment with the antioxidant N-acetylcysteine (NAC), suggesting involvement of oxidative stress (Fig 4A and 4B). Treatment with NAC in CS-exposed cultures partially restored wound repair (Fig 4C). In contrast, CS-induction of RNase 7 in wounded ALI-PBEC was completely inhibited by NAC (Fig 4D), and also CS-induced IL-8 mRNA expression was significantly inhibited (Fig 4E). These findings demonstrate a differential effect of CS-induced oxidative stress on wound repair and innate immune responses, which are suppressed and enhanced respectively.

### EGFR- and ERK1/2-dependent signaling are required for wound repair and innate immune responses

**Fig 5:**
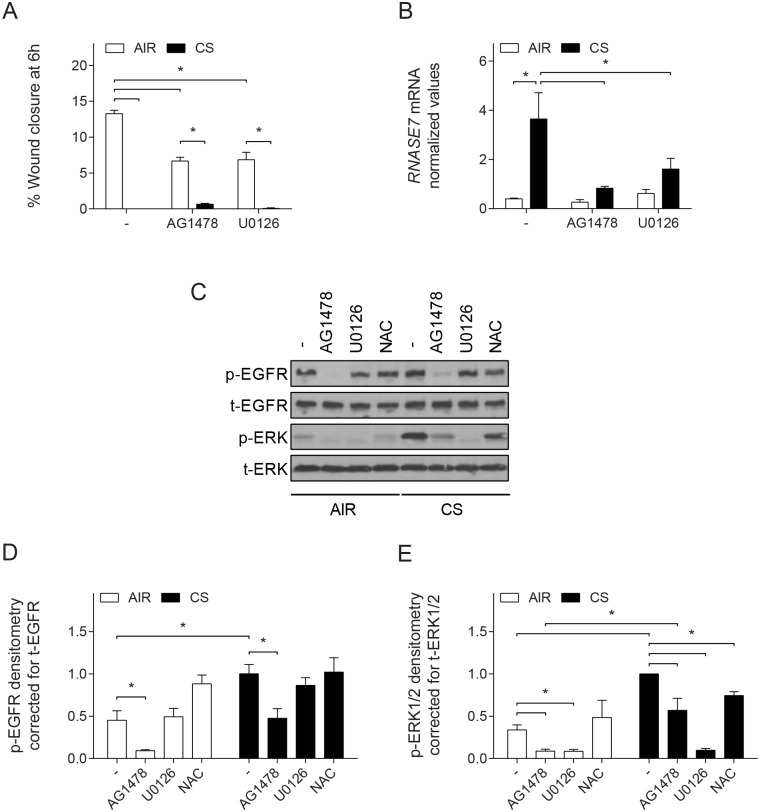
EGFR and ERK1/2 signaling in wounded ALI-PBEC. (A) Intrinsic wound healing of ALI-PBEC was determined in the presence of the EGFR tyrosine kinase inhibitor AG1478 (1 μM) or the MEK1/2 inhibitor U0126 (25 μM) at 6 h after exposure with either air or CS. Data are shown as percentage wound closure compared to t = 0 h. (B) mRNA expression of *RNASE7* was determined by qPCR. Data are shown as normalized mRNA expression compared to *RPL13A* and *ATP5B*. (C) Western blot analysis of EGFR and ERK1/2 phosphorylation of wounded ALI-PBEC exposed to air or CS in the presence of AG1478, U0126, and NAC, at 6 h after exposure. (D) Bands were quantified by densitometry for analysis of EGFR and (E) ERK1/2 phosphorylation and corrected for total-EGFR and total-ERK1/2, respectively. For all graphs data are shown as mean; error bars represent SEM; experiments were conducted in duplicate; n = 3 independent donors; * p < 0.05.

**Fig 6:**
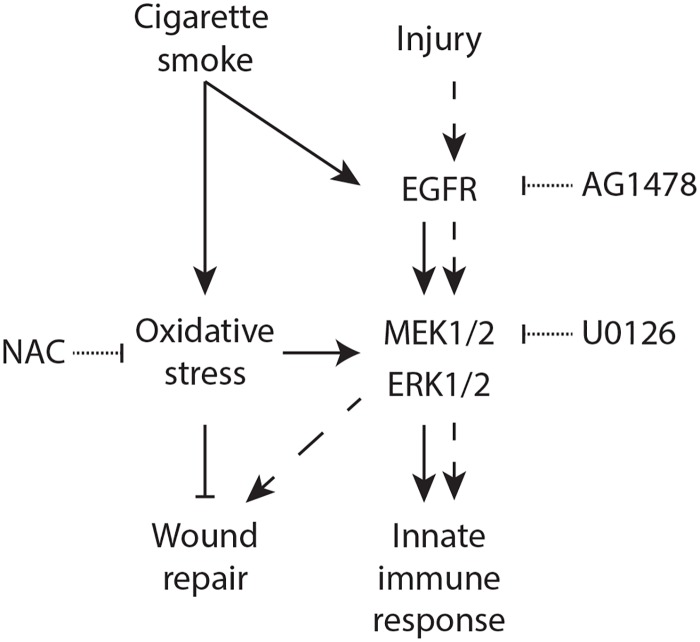
Proposed model. EGFR signaling is activated by CS and injury, and this leads to MEK1/2-mediated phosphorylation of downstream ERK1/2. CS furthermore directly causes phosphorylation and activation of ERK1/2 via oxidative stress, which is independent of EGFR signaling. EGFR/ERK1/2-mediated wound repair is suppressed by CS via oxidative stress. In contrast, activation of ERK1/2 due to a combined effect of CS-induced oxidative stress and injury, results in an enhanced innate immune response. Solid lines represent the effect of CS, dashed lines the effect of injury. NAC, AG1478 and U0126 were used to inhibit oxidative stress, EGFR phosphorylation, and ERK/12 phosphorylation respectively.

The epidermal growth factor receptor (EGFR) and downstream MAP-kinase/extracellular signal-regulated kinase (ERK)1/2 signaling pathway are important in both wound repair and induction of innate immune responses [10]. Therefore, we examined the role of EGFR and ERK1/2 in the repair and induction of innate immune responses in wounded ALI-PBEC. First, the contribution to the intrinsic wound repair of ALI-PBEC was demonstrated by inhibitor experiments, showing impaired wound healing in the presence of AG1478 (EGFR tyrosine kinase inhibitor) and U0126 (Mitogen activated protein kinase/ERK kinase (MEK)1/2 inhibitor) (Fig 5A). As CS completely impaired wound healing at 6 h, we did not observe an additional effect of EGFR or MEK1/2 inhibition. In agreement with our previous study [9], CS-induced mRNA expression of RNase 7 in wounded ALI-PBEC cultures was significantly inhibited upon EGFR and ERK1/2 inhibition (Fig 5B). We subsequently determined EGFR and ERK1/2 phosphorylation in wounded ALI-PBEC. Phosphorylation of both proteins was observed in CS-induced wounded ALI-PBEC (Fig 5C–5E). EGFR inhibition completely suppressed ERK1/2 phosphorylation in air-exposed cells, whereas in CS-exposed cells only a partial inhibition was observed. In contrast, inhibition of ERK1/2 phosphorylation did not affect EGFR phosphorylation. The contribution of CS-induced oxidative stress to EGFR and ERK1/2 phosphorylation was determined by examining the effect of antioxidant treatment. CS-induced EGFR phosphorylation in wounded ALI-PBEC was not altered by NAC (Fig 5C–5E), whereas NAC did decrease CS-induced ERK1/2 phosphorylation. This suggests an EGFR-independent activation of ERK1/2 mediated by oxidative stress. In summary, these findings indicate involvement of EGFR- and ERK1/2 signaling in intrinsic wound healing, and in CS-induced innate immune responses during repair. Moreover, CS increases ERK1/2 phosphorylation in both an EGFR-dependent and -independent pathway mediated in part by oxidative stress (Fig 6).

## Discussion

In this study we examined the effect of whole CS exposure on wound repair and innate immune responses of injured ALI-PBEC cultures. We observed a detrimental effect of acute CS exposure on the restoration of the epithelial barrier integrity and wound closure after mechanical wounding. In contrast, induction of innate immune responses, in particular expression of RNase 7, was further enhanced in CS-exposed injured ALI-PBEC. The impairment of epithelial repair in the mechanical injury model was accompanied by an accumulation of BCs at the wound edge. Moreover, oxidative stress contributed to the CS-induced attenuation of wound repair and the induction of innate immune responses. Both intrinsic wound repair and CS-induced innate immune responses required EGFR- and ERK1/2 mediated signal transduction.

Whole CS exposure of ALI-PBEC attenuated airway epithelial repair, in particular during the first 6 h following CS exposure. Such a transient effect of CS is in line with our previous study, in which CS was shown to transiently affect the epithelial barrier integrity followed by restoration within 24 h [9]. Oxidative stress contributed to the observed effects of whole CS exposure on epithelial wound repair, as NAC partly reversed the observed induction of an anti-oxidant response and impairment of epithelial wound repair. This is in line with our previous findings in undifferentiated (submerged cultured) PBEC, using an aqueous extract of cigarette smoke [7], and suggest that findings made in these less physiological conditions remain relevant.

In contrast to the suppressive effect of CS on epithelial repair, CS-exposure resulted in increased innate immune responses in injured airway epithelial cell layers. Following CS exposure, increased IL-8 secretion was observed in the calcium switch model, whereas increased expression of RNase 7 was detected in the calcium switch as well as the mechanical wound model. Previously, we reported cell type-specific expression of RNase 7 by BCs present in ALI-PBEC cultures [9]. The current observation further suggests that BCs are particularly affected upon injury of ALI-PBEC. Indeed, BCs are regarded as a heterogenic population including epithelial progenitor cell of the pseudostratified airway epithelium [22, 23], and it is assumed that BCs repopulate denuded wound areas through cell spreading and migration after injury [24]. In agreement with this, the majority of cells at the leading wound edge of injured ALI-PBEC stained positive for the BC marker p63. In line with earlier reports [7, 25], this could not be explained by increased proliferation, as only limited numbers of proliferating cells were observed at the wound edge as assessed by BrdU incorporation (*data not shown*). However, p63^+^ cells displayed smaller internuclear distances upon CS exposure, suggesting that spreading and migration of BCs is impaired. Further studies are needed to determine whether cigarette smoke specifically targets subpopulations of BCs.

We speculate that the transient effect of CS in our model reflects the acute effects of smoking on the airway epithelium that is in a process of repair. Normally, BCs of the airway epithelium will close denuded wound areas through cell spreading before starting cell proliferation [26]. However, primarily under the influence of oxidative stress caused by smoke exposure, the cells shift towards a different function, displaying reduced repair-promoting migratory activity but increased innate immune and cytoprotective anti-oxidant responses. The transient effect of CS on wound repair suggests recover of airway epithelial cells from mild damage that do not cause extensive cell injury and promote cell death. Indeed, CS exposure induced the expression of genes involved in the Nrf2-mediated antioxidant and survival response, which suggests that this response is involved in the restoration of wound repair following CS exposure. This mechanism may be impaired in COPD, since previous studies have reported attenuated Nrf2-dependent antioxidant responses in the bronchial tissues from COPD patients compared to non-COPD smokers [27, 28]. Moreover, it has been shown that COPD airway epithelial cells display reduced wound repair and epithelial barrier properties [29, 30]. Therefore, it can be speculated that an impaired oxidative stress response is related to epithelial dysfunction during COPD disease progression. It needs to be noted that our experimental design was adapted to mimic the effects acute cigarette smoke exposure, and not that of the repeated exposures that are typical form smoker’s lungs. Further studies are needed to explore such effects during repeated CS exposure. Although the airway epithelial cells start to recover from the effects of CS in both the calcium switch and wound repair model at 6 h after exposure, induction of RNase 7 in BCs persisted at later time points. In particular, in the calcium switch assay enhanced expression of RNase 7 was observed at 24 h after smoke exposure, when the epithelial barrier integrity had recovered. This suggests that BCs display innate immune responses after the epithelium has recovered from injury. Epithelial injury results in activation of the EGFR signalling pathways in BCs, which is important for both wound closure and RNase 7 expression.

We propose that the reduced wound repair activity of the airway epithelium upon CS exposure increases the susceptibility of the epithelium and underlying tissues to microbial colonization and infections. The increased expression of the antimicrobial RNase 7 by BCs that occurs in parallel with impaired wound repair might be a compensatory mechanism to provide a last-resort antibacterial defense against invading microbes. We did not study the host defence activity of CS-exposed and injured airway epithelial cells using functional assays. Therefore, further research is required to determine the additional effects of adding live microbes in our wound healing model, and the putative modulating effect of increased RNase 7 expression. Antimicrobial proteins and peptides such as RNase 7 display immunomodulatory and wound repair enhancing properties [31, 32]. These responses might contribute to the wound repair process but might also contribute to cell injury when these mediators are produced in high amounts and/or during prolonged periods. There is however currently no evidence for other activities of RNase 7, and therefore further research is required to demonstrate this. We used mechanical wounding of the epithelial layer by scraping, which is widely used in studies on repair but is obviously a less physiological relevant model of injury than e.g. bacterial or viral infection or repeated smoke exposure. An important advantage of the model is, however, that it allows creation of a defined wound and quantification of its repair. Another advantage is that it allows an analysis of the interaction between microbial infection or smoke exposure and wound repair.

Previously, we reported the importance of EGFR signaling in induction of innate immune responses by CS, which was mediated by downstream ERK1/2 activation [9]. Antioxidant treatment did not reduce EGFR signaling, but did decrease ERK1/2 phosphorylation, suggesting an EGFR-independent activation of ERK1/2 by oxidative stress (Fig 6). Thus, although EGFR-signal transduction is critical in airway epithelial wound repair and innate immunity [33], these findings demonstrate that other signaling transduction pathways contribute to repair and might be affected by CS exposure. Further research on CS effects on other repair pathways is required, and might also elucidate the differential regulation of epithelial wound repair and RNase 7 expression in mechanically wounded ALI-PBEC.

In summary, our findings demonstrate disturbances in the repair of injured airway epithelium and epithelial innate immunity upon cigarette smoke exposure. Oxidative stress caused by smoking is a key mechanism in modulating these responses, and in particular affects the activity of basal cells. These findings contribute to our understanding of how the repair and innate immune activity of wounded airway epithelial cells can be affected by cigarette smoking and might contribute to the development and progression of COPD.

## Supporting Information

S1 Fig Live imaging of intrinsic airway epithelial wound repair. (A) Primary bronchial epithelial cells (PBEC) were cultured and differentiated in an air-liquid interface (ALI) model, and subsequently mechanically wounded to assess wound repair. (B) The wound closure of ALI-PBEC was followed by live imaging at 0, 6, 12, 24, 36 and 48 h after wounding. (C) Wound closure was determined each hour, up to 48 h, by live imaging. Data are shown as the percentage wound closure compared to t = 0. Data are shown as mean; error bars represent SEM; experiments were conducted in duplicate. N = 3 independent donors.
(PDF)

S2 Fig Quantification methods of p63^+^ cells in wounded ALI-PBEC. (A) Analysis of p63^+^ and p63^-^ DAPI-stained nuclei at the leading wound edge. (B) Graphic example of how the internuclear distances were determined between p63^+^ cells located at the leading wound edge and p63^+^ cells that were perpendicular to the wound. p63^+^ cells at the leading wound edge were defined by the absence of other p63^+^ cells in the 45°-135° angle in its front perpendicularly to the wound edge. The most proximate p63^+^ cell that did not fulfill this definition was considered the reference cell to be selected for the measurement of the internuclear distance between adjacent p63^+^ cells. The distance between the outside edges of these two cells was regarded the internuclear distance. To prevent underestimation of distances in p63^+^ denser areas, each non-wound edge cell could be used only once for internuclear distance assessment, targeting overall at the lowest mean distance. The measurements were done in 5 randomly taken images of air- and CS-exposed ALI-PBEC. This analysis was performed in cultures derived from 3 independent donors.
(PDF)

S1 File Video of airway epithelial wound repair. (MOV)

S1 Table qPCR primer sequences. (DOCX)
