# Supplementary figures and images for: Cigarette Smoke Modulates Repair and Innate Immunity following Injury to Airway Epithelial Cells
Source: PLoS One. 2016 Nov 9;11(11):e0166255. doi: 10.1371/journal.pone.0166255 (PMC5102360; doi:10.1371/journal.pone.0166255)

A

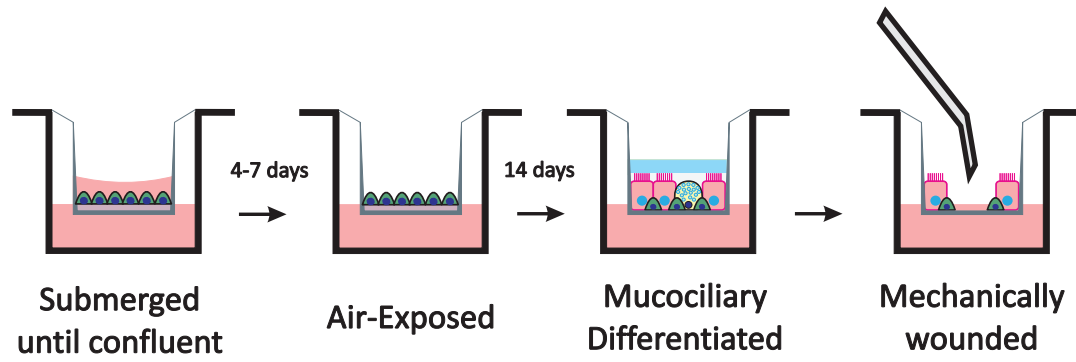

B

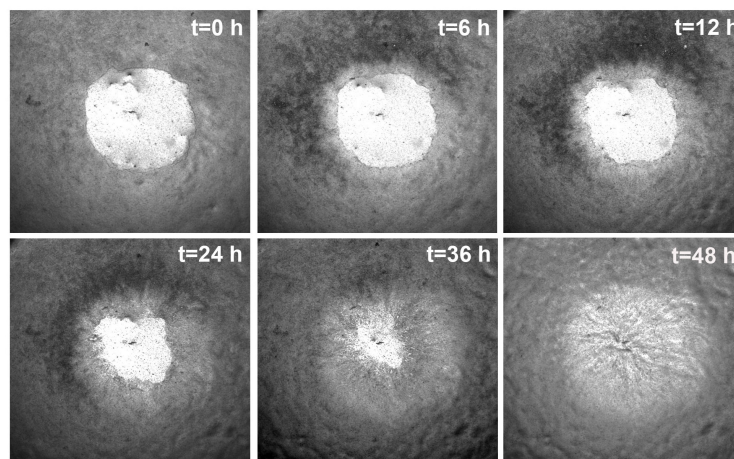

C

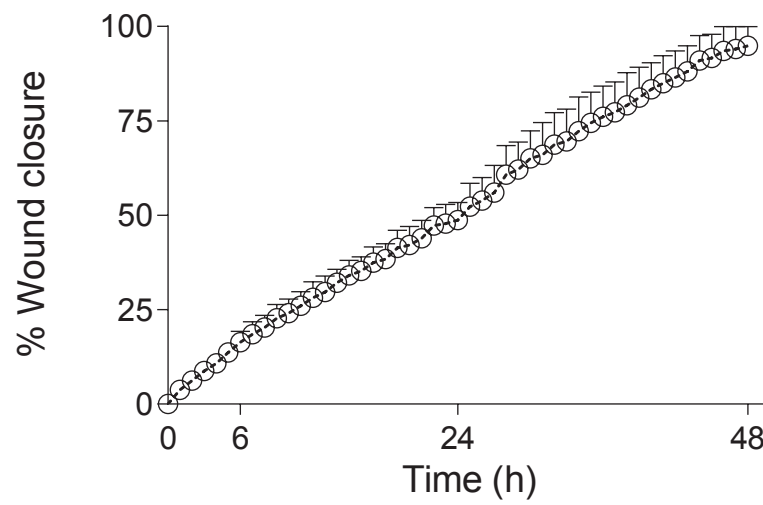

Supplement: S1 Fig — (A) Primary bronchial epithelial cells (PBEC) were cultured and differentiated in an air-liquid interface (ALI) model, and subsequently mechanically wounded to assess wound repair. (B) The wound closure of ALI-PBEC was followed by live imaging at 0, 6, 12, 24, 36 and 48 h after wounding. (C) Wound closure was determined each hour, up to 48 h, by live imaging. Data are shown as the percentage wound closure compared to t = 0. Data are shown as mean; error bars represent SEM; experiments were conducted in duplicate. N = 3 independent donors. (PDF) [file pone.0166255.s001.pdf]

A

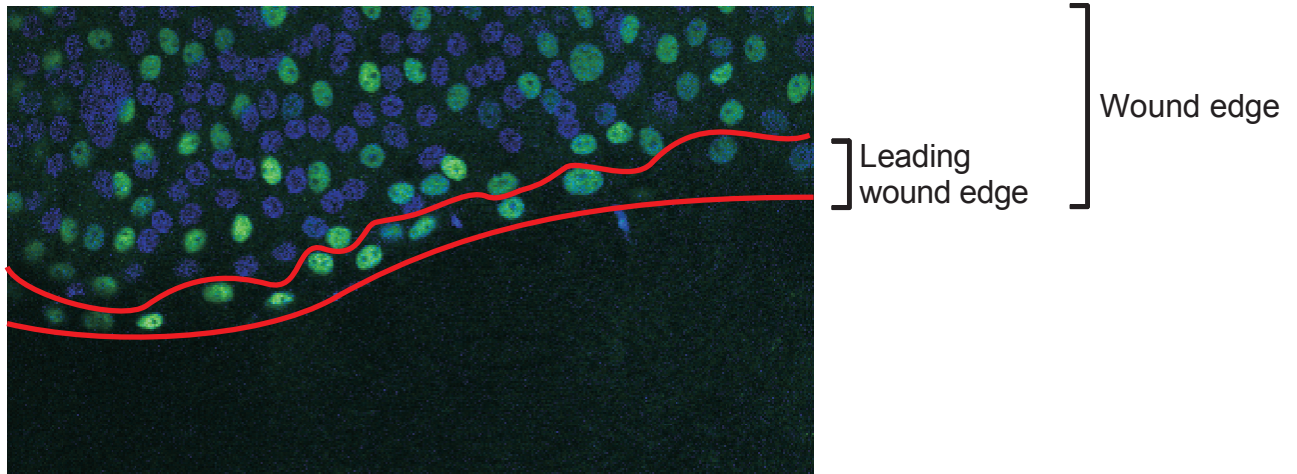

B Internuclear distances

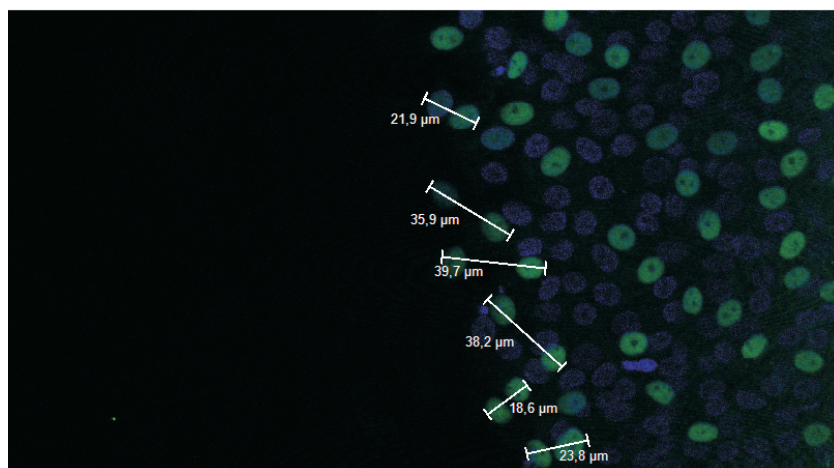

Supplement: S2 Fig — (A) Analysis of p63+ and p63- DAPI-stained nuclei at the leading wound edge. (B) Graphic example of how the internuclear distances were determined between p63+ cells located at the leading wound edge and p63+ cells that were perpendicular to the wound. p63+ cells at the leading wound edge were defined by the absence of other p63+ cells in the 45°-135° angle in its front perpendicularly to the wound edge. The most proximate p63+ cell that did not fulfill this definition was considered the reference cell to be selected for the measurement of the internuclear distance between adjacent p63+ cells. The distance between the outside edges of these two cells was regarded the internuclear distance. To prevent underestimation of distances in p63+ denser areas, each non-wound edge cell could be used only once for internuclear distance assessment, targeting overall at the lowest mean distance. The measurements were done in 5 randomly taken images of air- and CS-exposed ALI-PBEC. This analysis was performed in cultures derived from 3 independent donors. (PDF) [file pone.0166255.s002.pdf]
