# Supplementary material for: Cigarette Smoke Modulates Repair and Innate Immunity following Injury to Airway Epithelial Cells
Source: PLoS One. 2016 Nov 9;11(11):e0166255. doi: 10.1371/journal.pone.0166255 (PMC5102360; doi:10.1371/journal.pone.0166255)
Supplement: S1 Table — (DOCX) [file pone.0166255.s004.docx]

**S1 Table. qPCR primer sequences.**

| **Gene** | **Forward** | **Reverse** |
| --- | --- | --- |
| *HMOX1* | 5’-AACCCTGAACAACGTAGTCTGCGA-3’ | 5’-ATGGTCAACAGCGTGGACACAAA-3’ |
| *SCAL1* | 5’-GGCATTTACCAGCTGAGGGA-3’ | 5’-TACCCCTACCTAGCACAGCA-3’ |
| *RNASE7* | 5’-CCAAGGGCATGACCTCATCAC-3’ | 5’-ACCGTTTTGTGTGCTTGTTAATG-3’ |
| *IL8* | 5’-CAGCCTTCCTGATTTCTG-3’ | 5’-CACTTCTCCACAACCCTCTGC-3’ |
| *RPL13A* | 5’-AAGGTGGTGGTCGTACGCTGTG-3’ | 5’-CGGGAAGGGTTGGTGTTCATCC-3’ |
| *ATP5B* | 5’-TCACCCAGGCTGGTTCAGA-3’ | 5’-AGTGGCCAGGGTAGGCTGAT-3’ |
